# Military Surgery

**Published:** 1855-08

**Authors:** 


### ECLECTIC DEPARTMENT,

### AND SPIRIT OF THE MEDICAL PERIODICAL PRESS.

---

We cut from the N. Y. Daily Times the following report of a lecture by our friend Dr. Isidor Glück, of New York. Our September number will contain an elaborate article from his pen on Opacity of the Cornea. Dr. Glück has devoted much attention to the diseases of the eye, and though he does not make them a specialty in practice, his opinions are highly respected in the city of his adoption.

Those of our readers who see the American Medical Monthly, will recognize him as the author of a series of very valuable clinical lectures on diseases of the eye, which have appeared in that excellent journal.—*Ed. Buff. Med. Journal.*

*Military Surgery.—Lecture by Dr. Glück at the New York Medical College.*—The introductory to a course of lectures on this subject was commenced by Dr. Glück at the New York Medical College, in Thirteenth st., on Saturday, at 12 o'clock. In these times of arming and fighting it seemed to be particularly appropriate, and notwithstanding the heat we strolled up to listen. Dr. G. having served in the Hungarian army, drew most of his illustrations from the events of those campaigns, certainly not without an increase of sympathy in his hearers. He said, that however enthusiastic the surgeon may be, through innate love for his country, or other reasons touching him as a citizen, his duty as surgeon demands the utmost care for both *friend and foe alike*, hard as it may be, and really is, to divest himself of patriotic feelings. It is but natural that a soldier, if wounded, whether friend or foe, has reason to claim, by rights of humanity, equal attention and sympathy. Helpless as he is, he deserves our interest to the greatest extent, however unjust his cause, however wrong the motives that engaged or forced him to take up arms.

On the field of battle, as well as in the hospital, the severity of the case met with has to decide on the preference of attending, if friend and foe are mixed together and are equally accessible.

In the first instance, the sick or wounded soldier needs consolation, and encouragement that comes from the soldier's lips reäcts more beneficially when proffered in the soldier's native tongue.

Presence of mind, calmness, assurance, conservative boldness while operating, are attributes necessary to the military surgeon, who, above all, must possess a thorough medical education. Although the field of battle is the best medical school, it is so but to those who are not only perfectly familiar with the principles of surgery, but know at the same time *what* and *how to* observe, in order to be useful in the moment, and to turn it later to practical utility.

Military surgery differs from domestic or civil surgery in many points of view. Regardless of the severity of the wounds which mostly occur on the field, the circumstances of war aggravate the treatment, and those have to be consulted to a great extent; and indeed operations have often to be performed sooner, or modified and delayed altogether on that account. Whereas in domestic surgery the welfare of one has to be considered, the military surgeon must not lose sight of the influence on the plurality, and he is more justified in bestowing greater care upon those who—if soon discharged from the hospital—may render further services to the country or their cause, than to neglect them for the sake of trying "good luck" on absolutely hopeless cases; he should, however, not detract that attention that humane feelings dictate, even if death be imminent and certain.

The duties of a military surgeon are too numerous to be mentioned here, and mostly suggested by circumstances of war. These are in many respects different in time of peace from what they are during war. With slight differences, the duties of the military surgeon during peace are almost like those of a civil surgeon, while the duties during war are peculiar, and deserve therefore an especial and closer consideration.

Among the chief duties of the surgeon is recruiting—and middle-sized men of sufficient strength should be taken when there is a choice, and as near to this as possible under other circumstances.

No better proof is needed, as to how necessary it is to keep the troops in good spirits and enthusiastic, than the fact that it reflects beneficially on the new comers, who have to replace the sick and wounded, if not the dead ones. The 9th battalion—remarkable for its extraordinary heroism, called voros sapkás (red cap) noted, and not soon to be forgotten by many an Austrian and Croatian soldier, if he luckily escaped with one wound,—was the terror of the opposing armies, so much so that they were often brought one hundred miles or more, in carriages, in order to take a certain place of importance. Their *prestige* was so great, that by their presence it was calculated, not with probability, but assurance, the possession of a height, village or space to be gained, and the further plans arranged accordingly. Although the loss was always a great and terrible one—death almost sure to one part of them, on account of the great task to be executed—yet no battalion entered with more resignation, and did its duty, and were ready to die, than the 9th.

To give you an instance of the deliberate coolness of such young men as belonged to this battalion, I will briefly state to you the history of one case, to which I shall take opportunity to revert, on account of its peculiarity, when treating of gunshot wounds in the thigh. D. P——, a good looking red cap soldier, 24 years of age, received a wound in his left thigh, from a bullet discharged from a rifle of a barbarous serf, who stood on the top of a low church tower, and subsequently fell motionless at the feet of the wounded Honvéd. The red cap Honvéd, exasperated for the destruction of the enameled cross be wore in the pocket of his pantaloons, as a sacred reminiscence of his mother, fought like a lion, after having received the shot, alternately bayoneting, loading, and discharging. For his wound he little cared. The battle that began at two o'clock, and about four caused him a wound, found him at nine in the morning yet among the last on the field. Was it not for his cross, that he looked at immediately, and found an arm of it shattered, he would not have been aware of his wound. When the battle was at an end, he repaired to the ambulance with his swollen limb. The bullet could not be found, in spite of several attempts. The wound, however, healed up; after nine weeks, he wore his red cap again in rank, and was promoted to the rank of officer. He paid little attention to his leg, and it gave him no trouble while the war was raging. In his fortunate escape to England, his wound broke open, and passing some time in the hospitals of the various countries he traveled, the bullet was several times looked for, but not found,—he left the hospitals with the bullet in his thigh. While a refugee in London, he suffered off and on, until tired, when he resolved to have it cut off. One day much annoyed by the pain in his thigh, he called at my room, and not finding me in town, he applied to one of the surgeons of the day, who, after examination, could not ascertain the situation of the ball. Directing him, with a note to me, the surgeon requested me to assist him, and for that purpose to bring him to the hospital he had charge of. With this view I repaired with my countryman to the hospital, where, in the presence of the pupils, I explained that my comrade, aged twenty-four, had been in forty-nine engagements, and in one of these battles received a gunshot wound in his left thigh. A laugh of admiration by the pupils startled my comrade, who at that time ignorant of the English language, feared to appear ridiculous, and on no account would submit to any further trial. Walking home, the pain in his thigh increased, and he decided on having the ball cut out at once. He lit his cigar, ordered a pail of water, undressed, and laid down in bed. A penknife, used for cleaning his pipe, he seized for want of any other instrument, stuck it along the cruralis, two inches from the knee joint, and feeling with his finger, thrust it to the very bone, wherefrom he drew it upward, forming, most deliberately, a wound two inches in length, which admitted his finger to search for the ball, which he luckily extracted after some considerable searching. Bandaging his thigh, he applied cold lotions, and was well a fortnight later.

Accoutrements must be arranged as lightly as possible, ambulances provided, and portable kitchens, for the married soldier had rather go hungry than to cook. Recently a new model was devised by a late officer of the Hungarian army, and constructed in such a way as to prove more practical. It is on two wheels, and may be drawn by one or two horses in tandem; it can be easily carried over trenches, hills, and mountains. One man ascending behind, while on the way following the troops, may be continually preparing meals, so that the soldier may be served at any time indicated. The simplicity of construction would render it very valuable.

*Arms.* The surgeon's mission being to attend to the injuries inflicted by various weapons, it is necessary that he should be, if not familiar, at least somewhat acquainted with the arms in use—the more so as the surgeon himself may be called upon to use some of them in self-defence, if mixed up with the scuffle, as it often happened during the war in Italy, and particularly so in Hungary, where the surgeons wore the same uniform as the other officers in the army, with an additional white crape around the left arm, for which distinction the enemy little cared if he got hold of some of us.

As by the formation of a wound, the nature of the weapon, the projectile, its consistency, size, and form, the velocity and power with which it has been thrown, the angle under which the distance in which the missile was sent, has to be considered; and as further, the nature of the parts to be pierced, the elasticity of the textures and organs must be regarded, it must not be *superfluous* to enter into the construction and explain the properties of some arms now in use, before reverting to their various effects on the different parts of the body.

Here the Doctor exhibited all sorts of fire arms, and swords. Some of the Turkish being of a curious shape, curved in the opposite direction to that of the cutlass. Those people, however, do not try to cleave open a foe, but putting the weapon upon him, draw it, cutting a slice out if possible. Minie balls, Pritchard's balls, spherical, conical, &c., &c., were exhibited, but all seemed to have alike a wicked look. He went on to say:

In a revolution, or even regular war, it may happen that no time is left for drilling, and the immediate assistance of some part of the population being necessary, it naturally follows that the weapons most accustomed to will be used on such occasions. Thus Kosciusko achieved his first glorious victories mostly through the men provided with sickles. Guerilla bands were organized in Hungary to infest the enemy continually with the most different kind of arms and weapons.

Among the weapons used during the war was one kind exclusively handled with the greatest advantage by the regiment called Csikós. Those men came forward on Kossuth's powerful appeal of "the country is in danger." Although by nature tough, and leading, as cattle drivers, a solitary life, they are a set of men with the tinge of wild poetic feeling, and enjoy nothing more than melancholy plaintive songs, that strike peculiarly even *natives. En masse* they voluntarily repaired to the different recruiting places for being enlisted.

As no time was left for their being drilled, and in want of other arms and the necessary knowledge for using them, they fought with their own weapon, called "ostor," which consisted of a handle, measuring about one-half a yard, to which was attached a lash, from two to five yards long, terminating in a wire measuring from four to six inches. This weapon was used most effectually by them, to the greatest vexation of the Austrian cavalry, in a wild run on horseback, from a distance calculated to injure the face of the rider or of his horse, before he could use his arms, and to throw it around the enemy, who was unable to respond to such arms, not being accustomed to them, and thus to dismount him by strangulating some part of his body, and by a rapid movement to tear him from his horse. Often satisfied with the prize of the horse, they allowed the enemy to take to his heels, knowing that in some hospital he will remember them for their saluting him manually.

A hussar sword was soon substituted for this kind of weapon of their own, and their name, after having been drilled regularly, changed for Hunyady *Hussars.*

In conclusion, the lecturer stated his plan of proceeding. Though we did not understand when his next lecture was to be, we respectfully suggest that he take a cooler day for it, if he waits till September.

